# Adenosine A_2A_ receptor inhibition reduces synaptic and cognitive hippocampal alterations in Fmr1 KO mice

**DOI:** 10.1038/s41398-021-01238-5

**Published:** 2021-02-05

**Authors:** Antonella Ferrante, Zaira Boussadia, Antonella Borreca, Cinzia Mallozzi, Giorgia Pedini, Laura Pacini, Antonella Pezzola, Monica Armida, Fabrizio Vincenzi, Katia Varani, Claudia Bagni, Patrizia Popoli, Alberto Martire

**Affiliations:** 1 National Center for Drug Research and Evaluation, Istituto Superiore di Sanità, Rome, Italy; 2 Institute of Neuroscience (IN)-CNR, Milan, Italy; 3 Humanitas Clinical and Research Center – IRCCS, Rozzano, (MI) Italy; 4 Department of Neuroscience, Istituto Superiore di Sanità, Rome, Italy; 5 Department of Biomedicine and Prevention, University of Rome Tor Vergata, Rome, Italy; 6 Department of Morphology, Surgery and Experimental Medicine, Pharmacology Section, University of Ferrara, Ferrara, Italy; 7 Department of Fundamental Neurosciences, University of Lausanne, Lausanne, Switzerland; 8 Present Address: Saint Camillus International University of Health and Medical Sciences, Rome, Italy

**Keywords:** Molecular neuroscience, Hippocampus

## Abstract

In fragile X syndrome (FXS) the lack of the fragile X mental retardation protein (FMRP) leads to exacerbated signaling through the metabotropic glutamate receptors 5 (mGlu5Rs). The adenosine A_2A_ receptors (A_2A_Rs), modulators of neuronal damage, could play a role in FXS. A synaptic colocalization and a strong permissive interaction between A_2A_ and mGlu5 receptors in the hippocampus have been previously reported, suggesting that blocking A_2A_Rs might normalize the mGlu5R-mediated effects of FXS. To study the cross-talk between A_2A_ and mGlu5 receptors in the absence of FMRP, we performed extracellular electrophysiology experiments in hippocampal slices of *Fmr1* KO mouse. The depression of field excitatory postsynaptic potential (fEPSPs) slope induced by the mGlu5R agonist CHPG was completely blocked by the A_2A_R antagonist ZM241385 and strongly potentiated by the A_2A_R agonist CGS21680, suggesting that the functional synergistic coupling between the two receptors could be increased in FXS. To verify if chronic A_2A_R blockade could reverse the FXS phenotypes, we treated the *Fmr1* KO mice with istradefylline, an A_2A_R antagonist. We found that hippocampal DHPG-induced long-term depression (LTD), which is abnormally increased in FXS mice, was restored to the WT level. Furthermore, istradefylline corrected aberrant dendritic spine density, specific behavioral alterations, and overactive mTOR, TrkB, and STEP signaling in *Fmr1* KO mice. Finally, we identified *A*_*2A*_*R* mRNA as a target of FMRP. Our results show that the pharmacological blockade of A_2A_Rs partially restores some of the phenotypes of *Fmr1* KO mice, both by reducing mGlu5R functioning and by acting on other A_2A_R-related downstream targets.

## Introduction

Fragile X syndrome (FXS) is characterized by a complex clinical phenotype and symptoms that include hyperactivity, autism, attention disorders, and seizures^1–6^. The disease is caused by an expansion of CGG-repeats in the fragile X mental retardation 1 gene (*FMR1*) that encodes fragile X mental retardation protein (FMRP). As a result, the expression of the gene is impaired, and either partial or complete silencing of FMRP occurs^7^. FMRP is an RNA-binding protein^8^ that regulates different steps of mRNA metabolism^9–14^ and binds only a fraction of mRNAs expressed in the brain^15^. A primary role of FMRP in the brain is the control of synaptic plasticity by regulating mRNA metabolism and repressing protein synthesis in dendrites and synapses^12,13,16,17^, and thereby its lack results in excessive protein synthesis^18^. How FMRP impairment causes FXS is not yet fully understood but some mechanisms are considered particularly relevant. First of all, it has been proposed that over-activation of metabotropic glutamate 5 receptor (mGlu5R)-mediated signaling plays a causal role in FXS^19^. This “mGluR theory” was strongly supported by the finding that genetic reduction of mGlu5R expression is sufficient to correct a broad range of phenotypes in the *Fmr1* KO mouse, a murine model useful for the study of FXS^20^. Additionally, pharmacological studies have shown that short-acting mGlu5R inhibitors, such as MPEP and Fenobam, can ameliorate FXS phenotypes in several evolutionarily distant animal models^21^. Moreover, Michalon and colleagues^22^ showed that the potent and selective mGlu5R inhibitor CTEP can correct phenotype abnormalities in young adult *Fmr1* KO mice, after the development of the phenotype. Finally, clinical studies suggested possible effects of the mGlu5R antagonist AFQ056 only on a subset of FXS patients^23^. Despite all these promising studies, in clinical trials no clear therapeutic benefit has been confirmed in heterogeneous populations of FXS patients treated with different mGlu5R inhibitors^24,25^. Although the reasons for the lack of translation from the preclinical to the clinical setting are largely unknown, the broad age range (12–40 years) of FXS individuals in clinical trials might have prevented the detection of a therapeutic benefit, which is more likely in young children, with an ongoing neurodevelopment^26^. Consistently, a new NIH-funded clinical trial in which the effects of AFQ056 will be specifically evaluated on language learning in young children (32 months to 6 years) with FXS is on progress (https://www.fraxa.org/fx-learn-clinical-trial-is-enrolling-children-with-fragile-x/).

Furthermore, a factor which has been relatively neglected in preclinical and clinical studies to date is the development of tolerance to mGlu5R inhibitors, already observed for the mGlu5R antagonist MPEP after repeated administration at high doses in *Fmr1* KO mice^27^.

This is currently an object of investigation (https://www.fraxa.org/pharmacological-tolerance-in-the-treatment-of-fragile-x-syndrome/).

Adenosine is a naturally occurring purine nucleoside distributed ubiquitously throughout the body that acts through multiple G protein-coupled adenosine receptor subtypes^28,29^. The adenosine A_2A_ receptors (A_2A_Rs), which play a major role in the brain, are effective modulators of neuronal damage in various pathological situations, and both their activation and blockade can be neuroprotective in different experimental conditions^30–33^. Although A_2A_Rs are most abundant in the striatum, they are also present in the hippocampus, a brain region strongly affected in FXS, where they finely modulate synaptic transmission and excitotoxicity^34^.

We hypothesized that A_2A_Rs could have relevance in FXS since these receptors exert a strong permissive role on mGlu5R-mediated effects in different brain areas^35,36^, and could contribute to direct or indirect modulation of different signaling pathways overactive in FXS^37–42^.

In this study, we first assessed whether the functional A_2A_/mGlu5 receptors interaction was altered in *Fmr1* KO mice; then, we evaluated if pharmacological blockade of A_2A_Rs was beneficial in terms of synaptic functions, neurobehavioral phenotype, and signaling features in *Fmr1* KO mice. Finally, we explored the *A*_*2A*_*R* mRNA/FMRP interaction in the mouse brain. Our findings indicate that A_2A_Rs play a role in FXS and that A_2A_R antagonist can modulate mGlu5R synaptic signaling and ameliorate behavioral phenotypes in FXS mice.

## Materials and methods

Methods are described briefly below; see Supplementary information for detailed descriptions.

### Animals

All procedures were carried out according to the principles outlined in the European Communities Council Directive, 2010/63/EU, DL 26/2014, FELASA, and ARRIVE guidelines, and approved by the Italian Ministry of Health and by the local Institutional Animal Care and Use Committee (IACUC). The experiments were performed on male and female C57Bl/6 wild-type (WT) and C57Bl/6 *Fmr1* knock-out (KO) mice between 10 and 18 weeks of age; FMRP immunoprecipitation was performed on male FVB.129P2 WT and *Fmr1* KO mice at 3 and 4 weeks of age. A colony of C57Bl/6 *Fmr1* KO mice^43^ was established starting from breeding pairs of animals (Charles River). Sample sizes were chosen according to information obtained from pilot studies or published data^22^. No randomization was performed to allocate subjects in the study. However, we allocated arbitrarily the animals to different experimental groups. No blinding was performed except for dendritic spine evaluation.

### Drugs and treatment

CHPG [(RS)-2-Chloro-5-hydroxyphenylglycine sodium salt], ZM241385 [4-(2-[7-Amino-2-(2-furyl)[1,2,4]triazolo[2,3-a][1,3,5]triazin-5-ylamino]ethyl)phenol], DHPG [(RS)-3,5-Dihydroxyphenylglycine], and CGS21680 [4-[2-[[6-Amino-9-(N-ethyl-β-D-ribofuranuronamidosyl)-9H-purin-2-yl]amino]ethyl]benzenepropanoic acid hydrochloride] (Tocris Biosciences) were dissolved in dimethyl sulfoxide (DMSO).

Istradefylline [8-[(1E)-2-(2-(3,4-Dimethoxyphenyl)ethenyl]-1,3-diethyl-3,7-dihydro-7-methyl-1H-purine-2,6-dione] (Tocris Biosciences, hereinafter referred to as KW6002) was orally administered solubilized in the vehicle prepared according to Orr et al.^44^. The weight of the animals and the fluid intake were assessed three times a week, and the concentration of the solution was adjusted so that the drug intake was maintained at 4 mg/kg per day^44^.

### Electrophysiology experiments

Hippocampal slice preparation and recordings were performed as previously described^39^. Extracellular field excitatory postsynaptic potential (fEPSPs) were recorded in *stratum radiatum* of the CA1 upon stimulation of Schaffer collaterals, then acquired and analyzed with the LTP program^45^. One slice was tested per experiment. Slices were obtained from at least two animals for each set of the experiment. mGluR-dependent long-term depression (LTD) was obtained by applying to hippocampal slices the group 1-mGluR agonist DHPG, 100 μM for 5 min^46^. Maximal transient depression (MTD) for a slice was defined as the time point post-DHPG application with the greatest depression within each slice. The DHPG-induced LTD was expressed as the mean percentage variation of the slope 60 min after treatment.

### Spine number and morphological analyses

These experiments were performed on rapid Golgi-Cox-stained brain sections^47^. At the end of the chronic treatment with vehicle or KW6002, mice were deeply anesthetized and perfused transcardially with 0.9% saline solution (*n* = 3 animals per group). Brains were picked up and immediately immersed into a Golgi-Cox solution at RT for 6 nights. On the seventh day, brains were transferred in a 30% sucrose solution for cryoprotection and then sectioned with a vibratome. Coronal sections (100 μm) were collected and stained. Spine density was analyzed on hippocampal CA1 *stratum pyramidale* neurons. On each neuron, five 30–100 μm dendritic segments of secondary and tertiary branch order of CA1 dendrites were randomly selected^48^ and counted using Neurolucida software.

### Testis weight

Macroorchidism in male mice postnatal days (PND) 130 (18 weeks of age, immediately after 12 weeks of the vehicle or drug treatment) was assessed as previously described^22^.

### Behavioral evaluation

All behavioral tests were performed between 7:30 a.m. and 13:00 a.m. and after mice were acclimated to the behavioral testing room for at least 30 min. The following experimental groups were compared: WT VEH mice (*n* = 11; 6 males and 5 females), *Fmr1* KO VEH mice (*n* = 16; 8 males and 8 females), and *Fmr1* KO KW mice (*n* = 22; 12 males and 10 females). Open field, Novel object recognition (NOR), Marble-burying, and Rotarod tests were performed as previously described^49–52^.

### Protein extraction and western blot analysis

Hippocampal and cortical mouse tissue samples were homogenized in RIPA Buffer on ice, centrifuged at 12,000*g* for 20 min at 4 °C, and supernatants were stored at −80 °C. Equal amounts of proteins were separated on SDS-PAGE gel and transferred on a PVDF membrane (Biorad Laboratories). After blocking, membranes were incubated with specific antibodies (anti-Phospho-Erk1/2, anti-Erk1/2, anti-Phospho-mTOR, anti-mTOR, anti-TrkB, anti-BDNF, anti-β-actin) and consequently treated as detailed in the supplements.

### STEP activity/expression analysis

Crude synaptosomal fraction preparation and STEP activity evaluation were performed as previously described (Chiodi et al.^40^ and references therein). For the western blot analysis of STEP, samples (40 μg of proteins) were analyzed according to Mallozzi et al.^53^.

### FMRP immunoprecipitation

Brain extracts were prepared from cortex and hippocampus or striatum of WT and *Fmr1* KO male mice 3–4 weeks old, using RIPA buffer plus Protease inhibitor cocktail (Roche), Phosphatase inhibitor cocktails II and III (Sigma), 40 U/ml RNaseOUT (Invitrogen). Protein extracts were used for RNA-immunoprecipitation (RIP) using a specific anti-FMRP antibody^54^.

### Saturation binding experiments

Saturation binding experiments were carried out on homogenized striatum, cortex, and hippocampus from WT and *Fmr1* KO mice (2–3 months of age), by using the A_2A_R antagonist [^3^H]-ZM241385 as radioligand.

### Statistics

Results from experiments were expressed as mean ± standard error of mean (SEM). For details, see the description in results, legends, and supplements. Statistical analysis was performed by using Mann-Whitney test or Student’s *t*-test, and *p*-value < 0.05 was considered to indicate a significant difference. All statistical analyses and electrophysiology curve fittings were performed by using GraphPad Prism software (USA).

## Results

### A_2A_R differently modulates the hippocampal mGlu5R-induced synaptic effects in *Fmr1* KO mice

**Fig. 1:**
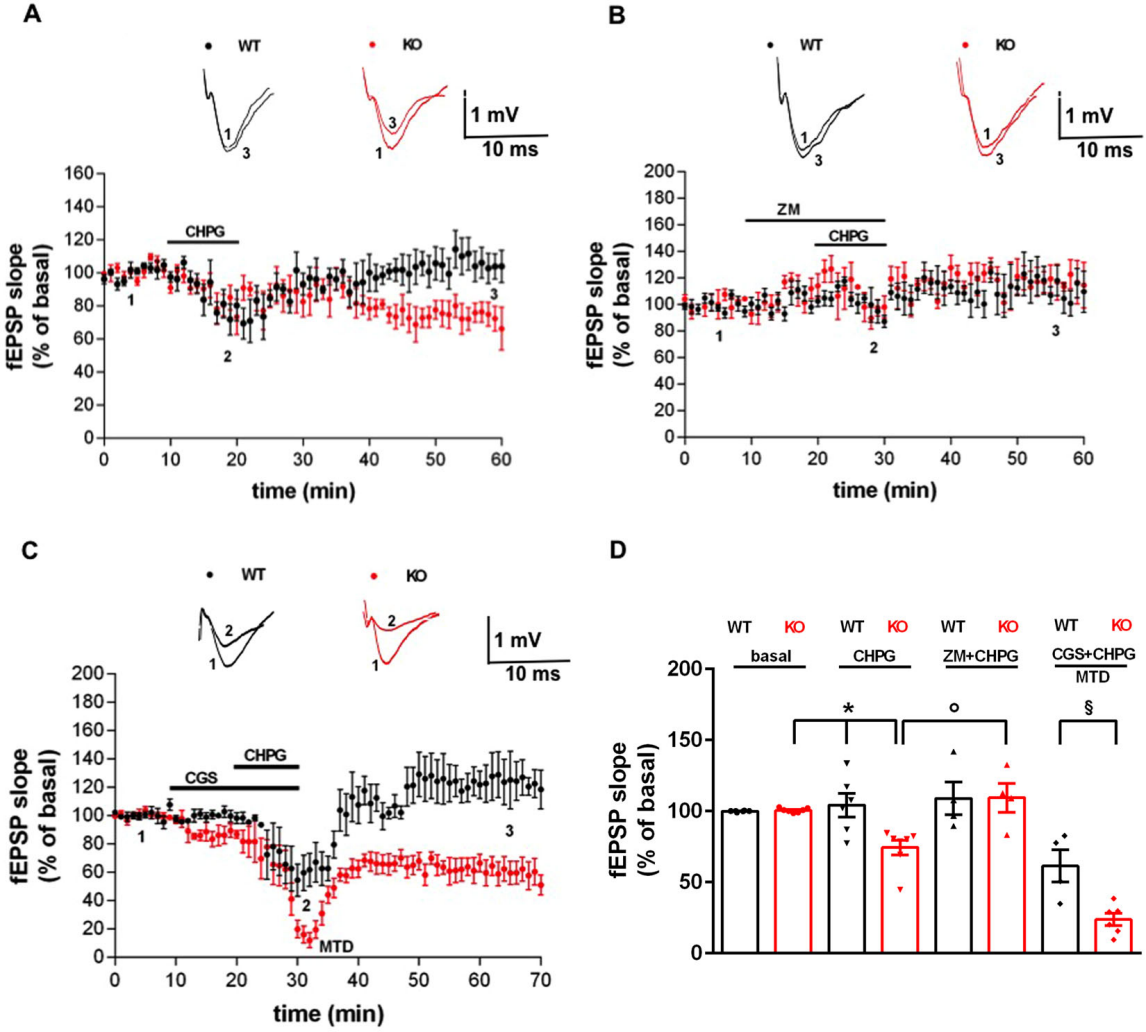
The CHPG-induced synaptic depression in *Fmr1* KO mice is modulated by A_2A_R ligands. **A** The selective mGlu5R agonist CHPG induced a long-lasting synaptic depression in the fEPSP recorded in *Fmr1* KO mice (*n* = 4 animals, 7 slices, *p < 0.01 vs. basal and *p < 0.05 vs. WT, Mann-Whitney test), while in WT mice (*n* = 5 animals, 6 slices) the synaptic transmission recovered completely after the CHPG application. **B** In the *Fmr1* KO mice, the long-lasting effect of CHPG on synaptic depression was prevented by a pre-treatment with the selective A_2A_R antagonist ZM241385 (*n* = 3 animals, 4 slices, °*p* < 0.05 vs. CHPG alone in *Fmr1* KO, Mann-Whitney test). **C** A pre-treatment with the selective A_2A_R agonist CGS21680 potentiated CHPG-induced MTD in *Fmr1* KO mice (*n* = 5 animals, 6 slices, ^§^*p* < 0.05 vs. CGS + CHPG in WT, Mann-Whitney test). **D** The dot-plot graph summarizes the results. Insets in panels (**A–C**) show representative fEPSP recordings in correspondence of baseline (1), MTD (2), or late washout (3). Calibration bars: 1 mV, 10 ms.

*Fmr1* KO mice and age-matched (10 weeks) control WT mice showed comparable basal transmission and paired-pulse facilitation, as previously reported^55,56^. We aimed at testing if A_2A_R ligands modulate the slight synaptic depression induced by the weak, selective mGlu5R agonist CHPG. In Fig. 1A we show that CHPG (300 μM over 10 min) induced a longer synaptic depression in the fEPSP recorded in *Fmr1* KO mice (74.39 ± 5.26% of basal slope after 30 min of washout, *n* = 4 animals, 7 slices; *p < 0.01 vs. basal and *p < 0.05 vs. WT, Mann-Whitney test, Fig. 1A, D), while in WT mice the synaptic transmission fully and immediately recovered after the CHPG application (104.20 ± 8.28% of basal slope after 30 min of washout, *n* = 5 animals, 6 slices). In *Fmr1* KO mice (Fig. 1B) the effect of CHPG on synaptic depression was prevented by the selective A_2A_R antagonist ZM241385 (ZM; 100 nM; 109.40 ± 10.13% of basal, *n* = 3 animals, 4 slices; °*p* < 0.05 vs. CHPG alone, Mann-Whitney test, Fig. 1B, D). Oppositely, the selective A_2A_R agonist CGS21680 (CGS; 100 nM; Fig. 1C) potentiated the CHPG-induced effect, particularly in terms of MTD (23.80 ± 4.33% of basal, *n* = 5 animals, 6 slices; ^§^*p* < 0.05 vs. CGS + CHPG in WT, Mann-Whitney test, Fig. 1C, D).

### KW6002 abolished the abnormal mGlu5R-dependent LTD in *Fmr1* KO mice

**Fig. 2:**
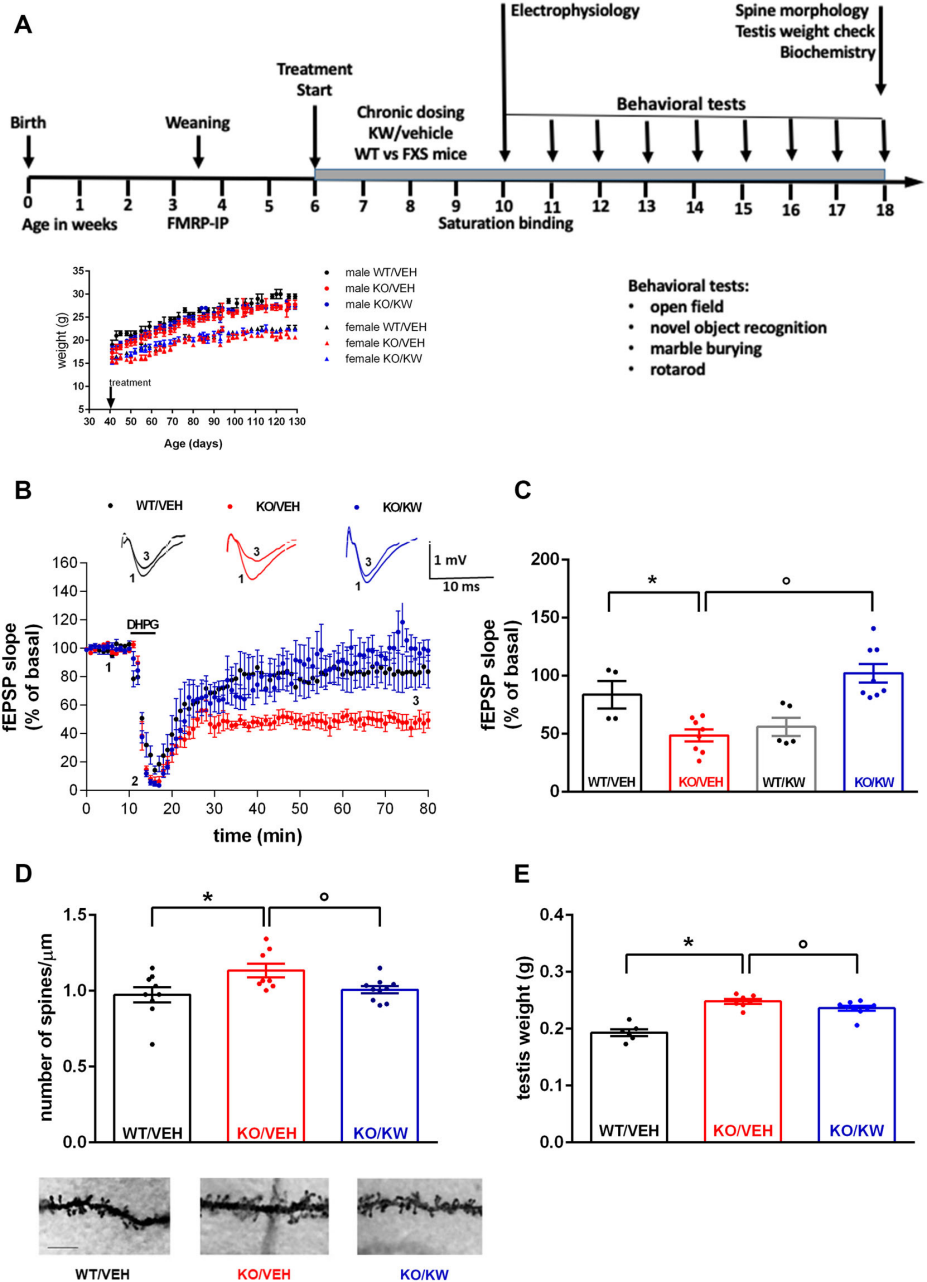
In vivo administration of the selective A_2A_R antagonist KW6002 reduces synaptic alterations and macroorchidism in *Fmr1* KO mice. **A** Timeline of chronic oral treatment schedule (4 mg/kg per day, PO) between 6 and 18 weeks of age. The inset shows that KW6002-chronic treatment did not affect body growth. The bodyweight increase during the 12 weeks of chronic treatment was not different between KW- and VEH-treated animals of both sexes. **B** The DHPG-induced LTD was strongly reduced in KO/KW mice (*n* = 4 animals, 8 slices, °*p* < 0.01 vs. KO/VEH, *n* = 5 animals, 8 slices, Mann-Whitney test). No difference was observed between WT/KW and WT/VEH mice (*n* = 2 animals, 5 slices, and *n* = 2 animals, 4 slices, respectively); a difference was observed between WT/VEH and KO/VEH mice (*p < 0.05, Mann-Whitney test). **C** The dot-plot graph summarizes the results. Insets in panel (**B**) show representative fEPSP recordings in correspondence of baseline (1) and late washout (3). Calibration bars: 1 mV, 10 ms. **D** Spine density was higher in dendrites of KO/VEH compared to WT/VEH mice (WT/VEH *n* = 9 and KO/VEH *n* = 8; *p < 0.05, Mann-Whitney test) and normalized in chronically treated KO/KW mice (*n* = 10, KO/KW vs. KO/VEH: °*p* < 0.05, Mann-Whitney test). The histograms show dendritic spines number (number of spines/dendrite segment length) counted on 5 segments per neuron in pyramidal neurons in the CA1 subfield of the hippocampus in WT and *Fmr1* KO mice, VEH- or KW-treated. Bars represent mean ± SEM. Representative images of Golgi-stained sections of the dorsal hippocampus and of apical dendrite segments of CA1 hippocampal pyramidal neurons (scale bar: 25 μm) in WT and *Fmr1* KO mice, VEH- or KW-treated, are reported below the graph. **E** KO/VEH mice (*n* = 7; 18 weeks of age) presented a larger testis weight compared to WT/VEH mice (*n* = 6; genotype effect: *p < 0.01, Mann-Whitney test), which was corrected in KO/KW mice (*n* = 9; treatment effect: °*p* < 0.05, Mann-Whitney test).

An orally active A_2A_R antagonist KW6002, or vehicle (the experimental groups are hereafter indicated as WT/VEH, WT/KW, KO/VEH, KO/KW), was administered to the mice. Electrophysiology experiments in hippocampal slices from *Fmr1* KO mice treated in vivo with KW6002 (from 6 weeks of age, for 4 weeks, 4 mg/kg per day, PO; Fig. 2A and inset inside showing that treatment did not affect body weight) were performed. In line with previous studies^46^, in KO/VEH mice the group 1-mGluR agonist DHPG (100 μM over 5 min) induced a clear LTD (48.45 ± 5.19% of baseline; *n* = 5 animals, 8 slices), which was larger compared to WT/VEH mice (*n* = 2 animals, 4 slices; *p < 0.05, Mann-Whitney test; Fig. 2B, C). Interestingly, the DHPG-induced LTD was strongly reduced in KO/KW mice, where fEPSP slope recovered to 102.10 ± 8.05% of baseline (*n* = 4 animals, 8 slices; °*p* < 0.01 vs. KO/VEH, Mann-Whitney test; Fig. 2B, C). No differences were observed between WT/KW and WT/VEH mice (Fig. 2C), except for a non-significant reduction in WT/KW group.

### KW6002 normalized hippocampal dendritic spine density and macroorchidism in *Fmr1* KO mice

Next we analyzed the spine number and morphology after KW6002 treatment. As shown in Fig. 2D, a significantly larger number of spines/μm was observed in the hippocampus of *Fmr1* KO vs. WT mice (WT/VEH: 0.974 ± 0.049, *n* = 9 neurons; KO/VEH: 1.134 ± 0.046, *n* = 8 neurons; *p < 0.05, Mann-Whitney test). Such an abnormal dendritic spine density was normalized in *Fmr1* KO mice treated with KW6002 over 4 weeks (KO/KW: 1.007 ± 0.023, *n* = 10 neurons; °*p* < 0.05 vs. KO/VEH, Mann-Whitney test). In addition, testis weight, as expected, resulted larger in KO/VEH mice in comparison to WT/VEH mice (respectively, 0.2481 ± 0.0042 g, *n* = 7 and 0.1932 ± 0.0060 g; *n* = 6; *p < 0.01, Mann-Whitney test). Macroorchidism was normalized by KW6002 in KO/KW mice (0.2361 ± 0.0042 g; *n* = 9; °*p* < 0.05 vs. KO/VEH, Mann-Whitney test; Fig. 2E).

### Chronic KW6002 corrects cognitive learning deficit in *Fmr1* KO mice

**Fig. 3:**
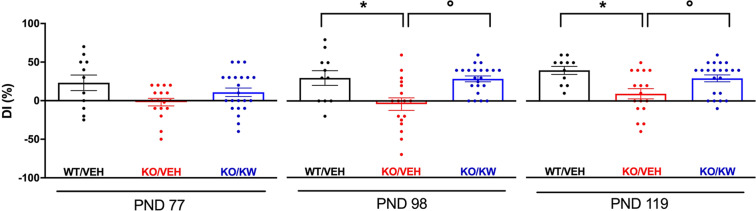
Effect of KW6002 on the preference for a novel object in *Fmr1* KO mice. In the NORT PND 98 KO/VEH mice exhibited a reduced ability to discern between old and new objects, as indicated by the lower DI in comparison to WT/VEH mice (*p < 0.05, Mann-Whitney test); chronic treatment with KW6002 induced an increase in DI in *Fmr1* KO mice (°*p* < 0.01, in comparison to KO/VEH, Mann-Whitney test); at PND 119 the lower DI of KO/VEH mice in comparison to WT/VEH mice (*p < 0.01, Mann-Whitney test) resulted increased after KW6002 treatment (°*p* < 0.05, in comparison to KO/VEH, Mann-Whitney test).

We then analyzed the cognitive learning in the *Fmr1* KO treated mice. In the NOR test (NORT), the time spent exploring the familiar and novel objects was quantified. We found that starting from PND 98 (Fig. 3) KO/VEH mice exhibited a reduced ability to discern between old and new objects as indicated by the lower value of discrimination index (DI: −4.12 ± 8.19 in KO/VEH, *n* = 16, vs. 30.00 ± 9.63 in WT/VEH, *n* = 11; *p < 0.05, Mann-Whitney test). Such impairment was attenuated by KW6002 (DI: 28.86 ± 3.84 in KO/KW; °*p* < 0.01 vs. in KO/VEH, Mann-Whitney test). The rescuing effect of KW6002 was maintained also at PND 119 (DI: 29.55 ± 4.48 in KO/KW, *n* = 22, vs. 9.41 ± 6.72 in KO/VEH, *n* = 16; °*p* < 0.05, Mann-Whitney test). KW6002 did not affect hyperactivity, stereotyped behavior, and motor coordination of mice (Supplementary Fig. S1).

### Effect of chronic KW6002 on mTOR signaling

**Fig. 4:**
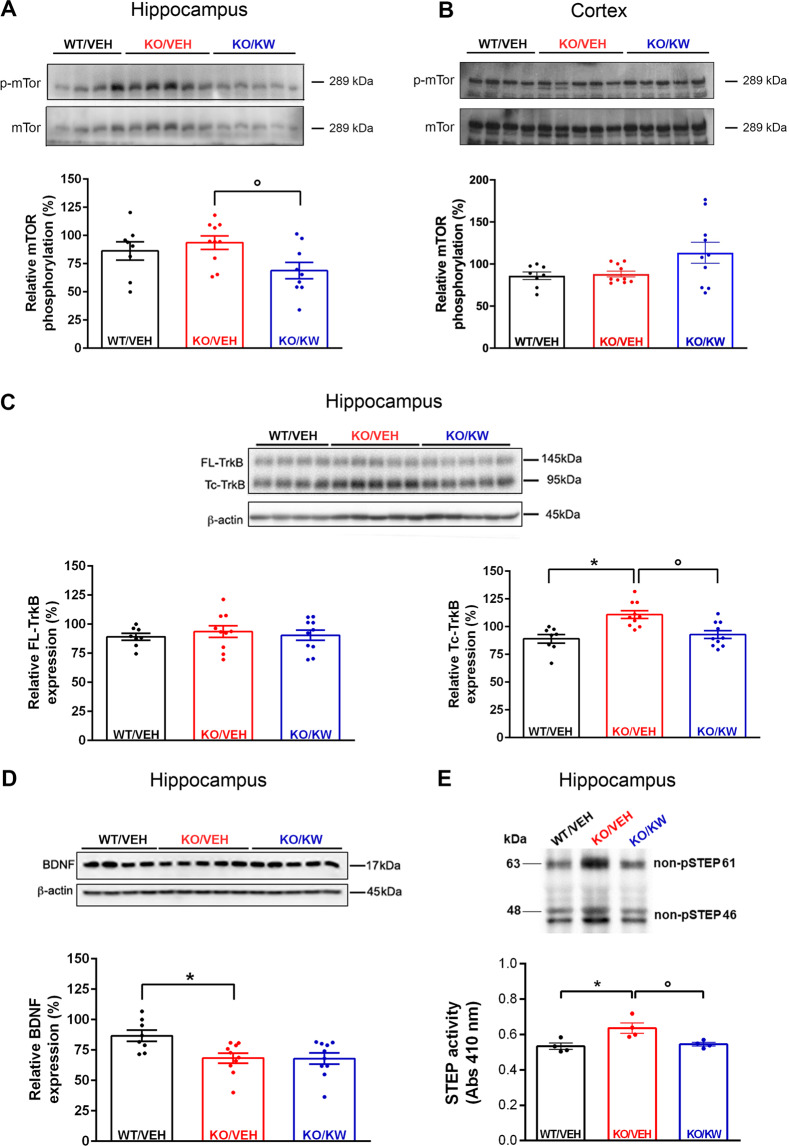
KW6002 modulates mTOR, TrkB, and STEP in the hippocampus of *Fmr1* KO mice. **A** Upper panel, representative western blot showing protein levels of mTOR and p-mTOR in the hippocampus. Each lane corresponds to tissue lysates from independent mice. Lower panel, histogram shows the quantification of the mTOR phosphorylation levels in KO/VEH mice (*n* = 10) compared to WT/VEH mice (*n* = 8; *p* = 0.46, Mann-Whitney test). mTOR phosphorylation level in the *Fmr1* KO mice upon chronic treatment with KW6002 (*n* = 9; °*p* < 0.05 vs. KO/VEH, Mann-Whitney test). **B** Upper panel, representative western blot showing protein levels of mTOR and p-mTOR in the cortex. Lower panel, histogram shows the quantification of mTOR phosphorylation in the different experimental groups. (**A, B**) the histograms indicate the % of phosphoproteins normalized to respective total protein levels. **C** Upper panel, representative western blot showing protein levels of FL-TrkB and Tc-TrkB in the hippocampus. Lower panel, quantification of FL-TrkB receptor level (left histogram) and Tc-TrkB receptor level (right histogram) in the hippocampus of KO/VEH (*n* = 10) in comparison to WT/VEH mice (*n* = 8; *p < 0.01, Mann-Whitney test). The treatment with KW6002 was able to correct this alteration in the Fmr1 KO mice (*n* = 10; °*p* < 0.01 vs. KO/VEH, Mann-Whitney test, right histogram); β-actin was used as normalizer. **D** Upper panel, representative western blot showing BDNF protein levels in the hippocampus. Lower panel, histogram shows reduced levels of mature BDNF in KO/VEH (*n* = 10; *p < 0.05, Mann-Whitney test) compared to WT/VEH mice (*n* = 8), but such a change was not affected by the KW6002 treatment (*n* = 10); β-actin was used as normalizer. **E** Upper panel, representative western blot showing protein levels of the two isoforms (61 and 46 kDa) of STEP protein. Lower panel, histogram shows an increased STEP activity in the hippocampus of KO/VEH compared to WT/VEH mice (*n* = 4; *p < 0.05, Mann-Whitney test). Chronic KW6002 treatment reduced significantly STEP activity in the Fmr1 KO mice (*n* = 4; °*p* < 0.05 vs. KO/VEH, Mann-Whitney test). The expression of the two isoforms (61 and 46 kDa) of STEP protein evaluated by western blot shows a similar trend (upper panel).

To investigate if A_2A_R blockade modulates one of the pathways found altered in FXS^22,38,57,58^, we analyzed mTOR (Fig. 4A, B) phosphorylation levels in hippocampus and cortex from WT and *Fmr1* KO mice treated with KW6002 or vehicle. In the hippocampus, the mTOR phosphorylation level was slightly increased in KO/VEH mice (93.66 ± 5.93; *n* = 10; Fig. 4A) compared to WT/VEH mice (86.32 ± 8.09; *n* = 8; *p* = 0.46 vs. KO/VEH, Mann-Whitney test). KW6002 reduced the mTOR phosphorylation level in *Fmr1* KO mice (68.83 ± 7.32; *n* = 9; °*p* < 0.05 vs. KO/VEH, Mann-Whitney test; Fig. 4A). No difference was detected in mTOR phosphorylation in the cortex (Fig. 4B). KW6002 did not reduce ERK1/2 hyper-phosphorylation found in the cortex of *Fmr1* KO mice (Supplementary Fig. S2b).

### Correction of TrkB and STEP signaling

We next analyzed a possible correction of other impaired molecular pathways in FXS. The expression of both full-length and truncated forms of TrkB receptor (FL-TrkB and Tc-TrkB, respectively; Fig. 4C), the mature form of BDNF (Fig. 4D), and STEP (activity and expression, Fig. 4E) were evaluated in the hippocampus of WT and *Fmr1* KO mice treated with KW6002 or vehicle. While no change was found in the FL-TrkB level (Fig. 4C, left panel), western blot analysis revealed a larger level of Tc-TrkB receptor in KO/VEH mice (110.80 ± 3.53; *n* = 10; *p < 0.01, Mann-Whitney test; Fig. 4C, right panel) in comparison to WT/VEH mice (89.09 ± 3.87; *n* = 8). KW6002 corrected this alteration in *Fmr1* KO mice (92.84 ± 3.47; *n* = 10; °*p* < 0.01 vs. KO/VEH, Mann-Whitney test; Fig. 4C, right panel). We observed a lower level of the mature BDNF form in KO/VEH mice, which was not affected by KW6002 (Fig. 4D). STEP activity was increased in KO/VEH mice (0.64 ± 0.03; *n* = 4; *p < 0.05, Mann-Whitney test; Fig. 4E) compared to WT/VEH mice (0.53 ± 0.04; *n* = 4) and KW6002 treatment reduced STEP activity in *Fmr1* KO mice (0.54 ± 0.01; *n* = 4; °*p* < 0.05 vs. KO/VEH, Mann-Whitney test; Fig. 4E). Consistently, STEP protein expression was also increased in KO/VEH mice and reduced by KW6002 treatment in *Fmr1* KO mice, as indicated by the western blot analysis performed by using an antibody that recognizes the two isoforms (61 and 46 kDa) of STEP (upper panel in Fig. 4E).

### A_2A_R mRNA is part of the FMRP complex

**Fig. 5:**
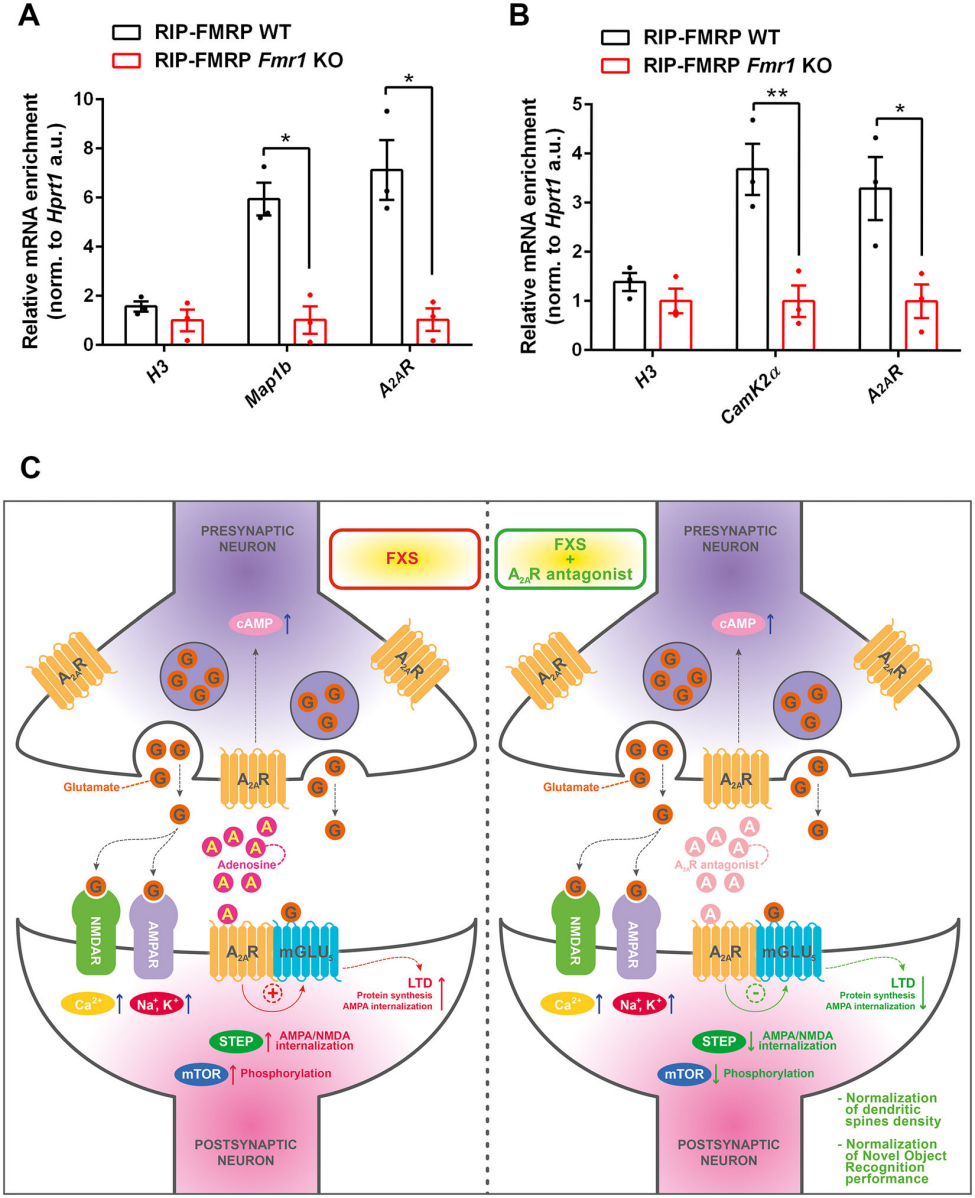
A_2A_R mRNA is part of the FMRP complex. The model summarizes the possible mechanisms of action due to A_2A_R blockade in Fmr1 KO mice. **A, B** *A*_*2A*_*R* mRNA is part of the FMRP complex. **C** Model summarizing the possible mechanisms of action due to A_2A_R blockade in *Fmr1* KO mice. (**A**) FMRP binds *A*_*2A*_*R* mRNA in cortex and hippocampus. *H3, Map1b*, and *A*_*2A*_*R* mRNAs were quantified by RT-qPCR. Shown is the enrichment of the immunoprecipitation/total, relative to *Hprt1* mRNA, mean ± SEM, *n* = 3; *p*-values were calculated by Student’s *t*-test (*p < 0.05). *Map1b* mRNA, a well-described FMRP target mRNA, was used as a positive control. (**B**) FMRP binds *A*_*2A*_*R* mRNA in the striatum. *H3, CamK2α*, and *A*_*2A*_*R* mRNAs were quantified by RT-qPCR. Shown is the enrichment of the immunoprecipitation/total, relative to *Hprt1* mRNA, mean ± SEM, *n* = 3; *p*-values were calculated by Student’s *t*-test (*p < 0.05; **p < 0.01). *CamK2α* mRNA, a well-described FMRP target mRNA, was used as a positive control. (**C**) In the left panel, the increased postsynaptic functional interaction between the A_2A_ and the mGlu5 receptors in *Fmr1* KO mice, could contribute to the aberrant mGlu5R-dependent LTD and mTOR hyper-phosphorylation in the FXS hippocampus. Here, the excessive STEP activity, which in turn contributes to AMPA internalization, could be directly ascribed to the aberrant A_2A_R signaling. A_2A_R abnormalities might derive from the defective FMRP complex control of *A*_*2A*_*R* mRNA translation at the early stages of mouse neurodevelopment. In the right panel, according to our data, the beneficial synaptic and cognitive effects deriving from A_2A_R blockade in the *Fmr1* KO mice could depend on the reduction of mGlu5R/mTOR and STEP activities. The TrkB/BDNF pathway could also partially contribute to it.

FMRP is an RNA-binding protein that regulates different aspects of mRNA metabolism^9–14^. To further investigate A_2A_R involvement in the FXS pathology we explored a possible association of FMRP to *A*_*2A*_*R* mRNA. To assess the presence of *A*_*2A*_*R* mRNA in the FMRP complex we performed an RNA immunoprecipitation with specific anti-FMRP antibodies^54^ using different brain regions from WT and *Fmr1* KO mice, followed by amplification of the associated mRNAs (Supplementary Table S1). Notably, we found that FMRP associates with *A*_*2A*_*R* mRNA in cortex and hippocampus (Fig. 5A). A_2A_Rs are mostly abundant in the striatum, consistently, we detected the associations of *A*_*2A*_*R* mRNA with FMRP also in this brain area (Fig. 5B). These findings suggest that FMRP could control *A*_*2A*_*R* mRNA metabolism leading to increased protein production.

## Discussion

In this study, we aimed to evaluate if A_2A_Rs play a role in FXS and if their blockade could modulate the physiological and behavioral phenotypes observed in the *Fmr1* KO mice. mGlu5Rs are under the tight control of A_2A_Rs in different brain areas^35,36,59–63^. As for the hippocampus, we demonstrated that A_2A_Rs need to be activated to allow the effects of mGlu5Rs to occur^36^. Here we investigated if the functional interaction between A_2A_R and mGlu5 receptors in the hippocampus was altered in the *Fmr1* KO mouse. We found that A_2A_R differently modulates the hippocampal mGlu5R-induced synaptic effects in *Fmr1* WT and KO mice. The ability of the A_2A_R agonist CGS21680 to potentiate CHPG-induced synaptic depression was found greater in *Fmr1* KO vs. WT mice, suggesting that the functional synergistic coupling between A_2A_ and mGlu5 receptors is larger in the absence of FMRP. We also tested if the chronic pharmacological blockade of A_2A_Rs restored the synaptic and behavioral phenotypes of *Fmr1* KO mice. We started by evaluating the LTD induced by the group 1-mGluRs agonist DHPG, and found that in vivo treatment with the A_2A_R antagonist KW6002 normalized the DHPG-LTD in *Fmr1* KO mice; this correction is likely due to enduring changes induced by chronic A_2A_R antagonism, different from the consequences of its acute blocking. Furthermore, we found that KW6002 reduced dendritic spine density in *Fmr1* KO mice. Although we do not know if such an effect could explain the LTD normalization, it is noteworthy that the colocalization of A_2A_ and mGlu5 receptors has been found mostly in synaptic contacts of cultured rat hippocampal neurons, where the A_2A_Rs are concentrated^36^.

In addition, we observed a reduction of learning deficit in the NORT behavioral paradigm. Such a beneficial effect exerted by KW6002 on the *Fmr1* KO mice might be the direct in vivo outcome of synaptic ameliorations of impaired LTD and dendritic spine density. It has been already demonstrated that synaptic plasticity, and particularly LTD, serves as a cellular substrate for the encoding of spatial object recognition^64^, and that also a non-genetic dendritic spine alteration (e.g. stress-induced reduction) is accompanied by a worsening in the NORT performance^65^. The efficacy of KW6002 in the cognitive domain is encouraging, considering that cognitive disabilities have the greatest impact on people living with FXS and their families^66^. Also, regarding the beneficial effect of KW6002 on the NORT performance, the inactivation of A_2A_Rs already proved to reduce cognitive dysfunctions in many different animal models useful to study neurodegenerative/neurological diseases^67–70^. After completing the behavioral tests, we assessed the possible impact of A_2A_Rs modulation on some measurable markers previously used to evaluate the preclinical potential of mGlu5R inhibitors^22^. According to the variability of mTOR phosphorylation described in the literature^38,57,58,71,72^, a slightly larger level of mTOR phosphorylation was detected in the hippocampus of the *Fmr1* KO, consistent with previous observations in the visual cortex^22^; interestingly, KW6002 was effective to reduce mTOR phosphorylation. Differently, the high variability in the signal of ERK1/2 levels prevented us from observing a clear effect of KW6002 on the hyper-phosphorylation found in the cortex of *Fmr1* KO mice. Besides the mGlu5R/mTOR modulation, we found that the A_2A_Rs directly impact other pathways altered in FXS, such as TrkB/BDNF and STEP^73^. Several studies suggest that TrkB/BDNF signaling pathway plays a role in FXS^41^, showing aberrances of BDNF and TrkB expression in murine brain lacking FMRP^74–76^. A_2A_Rs are required for the BDNF-induced potentiation of synaptic transmission in the mouse hippocampus^39^. In the present study, we found that the Tc-TrkB receptor level is increased in the hippocampus of *Fmr1* KO mice, and can be normalized by blocking the A_2A_Rs. Overexpression of Tc-TrkB receptor can inhibit full-length tyrosine kinase signaling^77–80^ and affect the control of dendritic morphology^81^. Notably, we found a decreased level of BDNF in the hippocampus of *Fmr1* KO mice, and the reduction of Tc-TrkB receptor in vivo partially rescues the phenotypes caused by loss of one BDNF allele^82^.

The absence of FMRP upregulates the translation of the mRNA encoding the STEP protein^42,83^. As a result, STEP levels are increased in the hippocampus of *Fmr1* KO mice^42^ and the pharmacological blockade^84^ or genetic deletion^42^ of STEP corrects *Fmr1* KO mice phenotypes. A_2A_Rs modulate the activity of this phosphatase^40^. An increased level of STEP activity is present in the striatum of A_2A_R-overexpressing rats and normalized by the A_2A_R antagonist^85^. Accordingly, in the present work, we confirmed that STEP activity is higher in the hippocampus of *Fmr1* KO mice and normalized by the A_2A_R antagonist.

Finally, we detected the presence of the *A*_*2A*_*R* mRNA in the FMRP complex in different brain areas of juvenile mice. These findings suggest a control of *A*_*2A*_*R* mRNA mediated by FMRP, possibly at the level of mRNA translation and at early stages of mouse neurodevelopment. Future investigation are necessary in the context of an unchanged affinity and density of the A_2A_Rs observed in adult *Fmr1* KO mice (Table S2), suggesting that A_2A_R abnormalities could start early in development and have an effect at later stages.

The beneficial effects deriving from A_2A_R blockade in *Fmr1* KO mice could depend on the contemporary reduction of mGlu5R/mTOR, TrkB/BDNF, and STEP activities, as summarized in the proposed model (Fig. 5C).

Considering the mGlu5R-relevant role in basal synaptic transmission and plasticity^86,87^, mGlu5R inhibitors might cause serious adverse events in humans^88^. Furthermore, treatment with mGlu5R inhibitors might likely result in tolerance development^27^, possibly being responsible for the disappointing outcome of the clinical trials^88^.

The use of the A_2A_R antagonists could help overcoming these limitations. Given the complexity of the molecular abnormalities observed in FXS^26,89^, targeting different pathways at the same time could represent a better therapeutic strategy.

The potential reliability of KW6002 is supported by pharmacodynamics/pharmacokinetics properties^44^ and safety^90^, being already used for Parkinson’s disease.

In conclusion, although further studies are needed to clarify the cross-talk between A_2A_ and mGlu5 receptors in the FXS condition, these findings suggest that A_2A_R antagonists could represent a possible novel therapeutic tool in FXS.

## Supplementary information

Supplemental material.
