# Supplementary material for: Adenosine A2A receptor inhibition reduces synaptic and cognitive hippocampal alterations in Fmr1 KO mice
Source: Transl Psychiatry. 2021 Feb 5;11:112. doi: 10.1038/s41398-021-01238-5 (PMC7864914; doi:10.1038/s41398-021-01238-5)
Supplement: Supplementary file 1 — Supplemental material. [file 41398_2021_1238_MOESM1_ESM.pdf]

Translational Psychiatry – Supplementary Information

Ferrante et al.

Supplementary Materials and Methods

Animals

All animal procedures were approved by the Italian Ministry of Health and by the local Institutional Animal Care and Use Committee (IACUC) at Istituto Superiore di Sanità (Rome, Italy; protocol number: 630/2017-PR) and University of Tor Vergata (Rome, Italy; protocol number: 1138/ 2016-PR, 494/2017-PR). FMRP-Immunoprecipitation experiments were performed on WT and *Fmr1* KO FVB.129P2 (CAT# JAX: 004624) male mice at postnatal day (PND) 21-28. Electrophysiology, biochemistry, dendritic spine morphology, and behavioral experiments were performed on male and female C57Bl/6 mice (CAT# JAX:000664, RRID: IMSR_JAX:000664; 25.50 ± 0.43 g weight for males at 10 weeks; 20.75 ± 0.48 g weight for females at 10 weeks), and on male and female C57Bl/6 *Fmr1* KO mice (CAT# JAX: 003025; 24.44 ± 0.46 g weight for males at 10 weeks; 20.27 ± 0.31 g weight for males at 10 weeks), between 10 and 18 weeks of age, unless otherwise specified. In this case, an in-house colony of C57Bl/6 *Fmr1* KO mice was established starting from breeding pairs of animals (The Jackson Laboratory, USA, by Charles River, Italy). *Fmr1* KO mice were bred by mating homozygous $Fmr1^{-/-}$ females and hemizygous $Fmr1^{-/y}$ males. Age-matched C57Bl/6 WT mice were used as control. No randomization method was used to allocate subjects in the study. We allocated arbitrarily the animals to the different experimental groups for *in vitro* or *ex vivo* evaluations, or the *in vivo* treatment with the A_{2A} antagonist istradefylline and the following behavioral test. The animals were kept in standard cages (48 x 26 x 20 cm, 4 mice per cage) under standardized temperature (22°C), humidity (55%), and lighting conditions (12:12 h light:dark cycle, with lights on at 6 am and euthanasia/withdrawn of organs and in vivo experiments conducted as soon as possible after the start of the light phase; usually, behavioral experiments were conducted between 7:30 am and 13:00 am) with free access to water and food. For animals used in the present study, proper treatment, care, and humane conditions have been provided. All efforts were made to reduce the number of animals used and to minimize their pain and discomfort. Permanent veterinary surveillance and animal welfare evaluation have been provided by the Host Institutions. As for behavioral experiments, we did not apply any inclusion or exclusion criteria, and no animals died during experiments.

Drugs and treatment

CHPG (mGlu5R agonist, Cat# 1049), ZM241385 (A_{2A}R antagonist, Cat# 1036), DHPG (mGluR agonist, Cat# 0342), CGS21680 (A_{2A}R antagonist, Cat# 1063), and istradefylline (A_{2A}R antagonist, Cat# 5147) were purchased from Tocris Biosciences (Bristol, UK). CHPG, ZM241385, CGS21680, and DHPG, were dissolved in dimethyl sulfoxide (DMSO) or in distilled water to obtain stock solutions. Stock solutions were made to obtain concentrations of DMSO lower than 0.001% in the superfusing ACSF and cell cultures. This DMSO concentration did not affect basal synaptic transmission in hippocampal slices (data not shown). Istradefylline (hereinafter referred to as KW6002), a selective adenosine A_{2A}R antagonist (permeable to the blood-brain barrier), was orally administered solubilized in the vehicle (the drug stock was prepared in 40% DMSO, 30% Cremophor EL and 30% mineral oil, and further diluted in 2% sucrose in water to prepare the final treatment solutions, which contained 0.2% DMSO, 0.15% Cremophor EL, and 0.15% mineral oil), in light protected bottles, and was continuously available. Istradefylline levels in the plasma and brain of mice exposed to similar doses and treatments have been accurately evaluated in [9]. The weight of the animals and the volume intake were assessed three times a week and the concentration of the solution adjusted so that the drug intake was maintained at 4 mg/kg per day. The control groups of mice were treated only with the vehicle.

Electrophysiological experiments

Slice preparation and recordings

Hippocampal slices were prepared as follows. After the sacrifice of animals by cervical dislocation, the hippocampus was removed and 450-μm slices were cut with a McIlwain tissue slicer (Cat # TC752, The Mickle Lab, Guildford, United Kingdom). Slices were maintained at RT (22–24°C) in ACSF containing (in mM): 126 NaCl, 3.5 KCl, 1.2 NaH_2PO_4, 1.2 $MgCl_2$, 2 $CaCl_2$, 25 $NaHCO_3$, 11 glucose (pH 7.3) saturated with 95% O_2 and 5% CO_2. After incubation in ACSF for at least 1 h, a single slice was transferred to a submerged recording chamber and continuously superfused at 32–33°C with ACSF at a rate of 2.6 ml/min. The drugs were added to this superfusion medium. The perfusion apparatus was made of chemically inert materials (silicone tubing). Extracellular fEPSPs were recorded in *stratum radiatum* of the CA1 hippocampus with a glass microelectrode filled with 2 M NaCl solution (pipette resistance 2–5 MΩ) upon stimulation of Schaffer collaterals with an insulated bipolar twisted NiCr electrode (50 μm OD). Each pulse was delivered every 20s (square

pulses of 100 μs duration at a frequency of 0.05 Hz), and three consecutive responses were averaged. The stimulation intensity used in the fEPSP recordings was always adjusted to obtain a submaximal fEPSP slope (~60% of maximum) with minimum population spike contamination. Signals were acquired with a DAM-80 AC differential amplifier (WPI) and analyzed with the LTP program. At least 10 min of stable baseline recording preceded drug application. Data were expressed as mean ± SEM of n experiments (one slice tested per experiment. Slices were obtained from at least two animals for each set of the experiment). To allow for comparisons between different experiments, slope values were normalized, taking the average of the baseline values to be 100%. The drug effect was expressed as the mean percentage variation of the slope from baseline over the last 5 min of drug perfusion. The washout period lasted at least 30 min.

Spine number and morphological analyses

The following protocol was used for the analysis of the spine number and morphology[1]. In brief, coronal brain sections were stained through consecutive steps in water (1 min), ammonium hydroxide (30 min), water (1 min), developer solution (Kodak fix 100%, 30 min), and water (1 min). Sections were then dehydrated through successive steps in alcohol at rising concentrations (50%, 75%, 95%, and 100%) before being closed with slide coverslips. Neurons were identified with a light microscope (Leica DMLB) under low magnification (20×/NA 0.5). Representative neurons within both hemispheres have been taken into consideration from each animal. Only protrusions with a clear connection of the head of the spine to the shaft of the dendrite have been counted as spines. Statistical comparisons were made on single neuron values obtained by averaging the number of spines counted on segments of the same neuron.

Behavioral evaluation.

Locomotor activity. The open field test was used to assess both locomotor activity and anxiety-related response. It was performed in a Plexiglass box (43x43x20cm) equipped with an overhead camera associated with the Anymaze video tracking system (Cat# 60000, UGO BASILE); the arena was arranged into a 16 square grid in the camera window with 4 squares in the center and 12 squares around the perimeter. Mice performed the test at PND 76, 97, and 118 for 20 min, and distance traveled in the center 4 squares (a measure of anxiety) and total distance (a measure of locomotion)

were compared between genotypes. Also, the center distance/total distance ratio was used as an index of anxiety.

Learning impairment. The *Novel object recognition test* (NORT) was performed as follows. The day after the open field test mice underwent the NORT: they were placed in the box facing the wall at the opposite end from the objects and were allowed to explore two identical objects located equidistant from the walls and the center of the open field for 10 min. One hour later, the trial was repeated with one of the objects being replaced with a novel object of similar size. The time spent exploring the familiar and novel objects was quantified. A mouse was considered to be exploring the object if it was sniffing, touching, or facing it within 2 cm or less, and measurements were recorded in seconds. Between each trial, the open field was cleaned to eliminate olfactory cues. A discrimination index (DI) was calculated as follows: ((time spent with the novel object − time spent with the old object)/ (time spent with the novel object + time spent with familiar object) × 100). Given the mathematical formula positive or negative DI values can be obtained. Time spent exploring each object was recorded by using the Any-maze video tracking system.

Stereotyped behavior. The effect of treatment with KW6002 on stereotyped behavior was evaluated in marble-burying tests at PND69, 82, 97, 112, 126. Mice were placed into a standard mouse cage containing clean corncob bedding (approximately 5 cm deep) and 20 marbles arranged in a 4 × 5 array on top of the bedding and left undisturbed for 30 min. At the end of the test, mice were carefully removed and the number of marbles that were more than 50% buried was recorded.

Rotarod test. To evaluate if chronic treatment with KW6002 could affect motor coordination and skill learning of animals the accelerating rotarod test was performed. To this aim, animals were trained on a rotarod apparatus (UGO BASILE) four times a day on two consecutive days with a constant speed of 12 rounds per min for 120 s. During the test, the rotation was increased from 4 to 40 rounds in 30 s steps within 5 min, and latency to fall was automatically recorded (in seconds). The test was performed once weekly starting from PND68.

Protein extraction and Western blot analysis.

Hippocampal and cortical mouse tissue samples were homogenized in RIPA Buffer containing 1% (w/v) Triton X-100, 0.5% (v/v) sodium deoxycholate, 0.1% (v/v) sodium dodecyl sulfate (SDS) in 1X PBS, with freshly added protease and phosphatase inhibitor (Thermo Scientific, Milan, Italy), and kept on ice for 30 min. After the incubation they were centrifuged at 12.000 × g for 20 min at +4 °C; the pellet was discarded and the supernatant transferred into new tubes and stored at -80 °C. Protein content was determined using the BCA protein Assay (Thermo Scientific, Bremen, Germany).

Protein extracts (5µg for ERK and 50µg for mTOR, TrkB, and BDNF) were resuspended in Laemmli loading buffer, heated for 5 min at 100 °C, and separated on SDS-PAGE gel. Proteins were transferred on a PVDF membrane (cat# 162-0177, Biorad Laboratories, Italy) with a 0.2 µm pore size, which was then blocked in 5% nonfat dry milk (cat# 170-6404, Biorad Laboratories, Italy) in T-TBS for 1h at RT. After blocking, membranes were incubated with: anti-Phospho-Erk1/2 (Thr202/Tyr204) monoclonal antibody (1:3.000, cat#4370, Cell Signaling Technologies, Danvers MA, USA), anti-Erk1/2 monoclonal antibody (1:3.000, cat#4695, Cell Signaling Technologies, Danvers MA, USA), anti-Phospho-mTOR (Ser2448) polyclonal antibody (1:1.000, cat#2971, Cell Signaling Technologies, Danvers MA, USA), anti-mTor polyclonal antibody (1:1.000, cat#2972, Cell Signaling Technologies, Danvers MA, USA), anti-TrkB monoclonal antibody (1:1000, cat#610101, BD Transduction Laboratories, Milan, Italy), anti-BDNF polyclonal antibody (1:1000, cat#PA5-85730, Invitrogen, Monza, Italy), anti-β-actin monoclonal antibody (1:5000, cat#MA5-15739, ThermoFisher Scientific, Milan, Italy), in 5% BSA (for anti-Phospho-Erk1/2, anti-Erk1/2, anti-Phospho-mTOR, and anti-mTor) or in 5% non-fat dry milk (for anti-TrkB, anti-BDNF, and anti-β-actin), and then in TBST at +4 °C. After incubation, membranes were washed three times in T-TBS for 10 min at RT, and incubated for 1h with anti-mouse horseradish peroxidase-conjugated antibodies (1:10.000, cat#115-035-003, Jackson Immunoresearch Laboratories, Euroclone, Italy), or anti-rabbit horseradish peroxidase-conjugated antibodies (1:10.000, cat#111-035-003, Jackson Immunoresearch Laboratories, Euroclone, Italy) at RT. Reactivity was detected using Western Bright ECL spray device (cat#K-12049-D50, Advansta, Aurogene, Italy) and Fluorchem, ProteinSimple (San Jose, CA, USA). Densitometric analysis was performed by using ImageJ software (http://rsb.info.nih.gov/ij/).

STEP activity/expression analysis

The crude synaptosomal fraction was prepared as follows according to the published protocol[2]. Briefly, the dissected hippocampus samples were homogenized in ice-cold buffer containing (in mM): 320 sucrose, 5 Hepes-NaOH, pH 7.4, 0.5 EDTA, 0.1 phenylmethylsulphonyl fluoride (PMSF) and protease inhibitor cocktail (cat# 05892970001, Complete, Roche) using a Teflon-glass grinder, by applying approximately 10-15 strokes and in any case up to the complete disintegration of the tissue sample. The homogenates were centrifuged at 800 g at 4°C for 10 min, and the supernatants were collected and centrifuged at 9200 g for 15 min. Pellets were washed in homogenization buffer and centrifuged at 10200 g for 15 min to obtain a crude synaptosomal fraction. Protein content was determined using the bicinchoninic acid assay (cat# 23227, BCA kit; Thermo Scientific, Waltham, MA, USA). Hippocampal crude synaptosomes were solubilized by incubation for 30 min at 0°C with

RIPA buffer (in mM): 100 Tris-HCl, pH 7.5, 600 NaCl, 4% (w/v) Triton X-100, 4% (v/v) sodium deoxycholate, 0.4% (v/v) SDS, 0.4 PMSF, and protease inhibitors (Complete, Roche). After centrifugation at 16000 g for 30 min at 4°C, the supernatant at a final concentration of 1.5 mg/ml was incubated with 50% (w/v) protein A/G PLUS agarose beads (cat#. sc-2003 from Santa Cruz Biotechnology, Santa Cruz, CA, USA) for 2 h at 4°C, clarified by centrifugation and incubated overnight at 4°C in a rotating wheel with a polyclonal anti-STEP antibody (cat# 4817, RRID: AB_2173544, 5 μg/sample). The immunocomplex was precipitated by the addition of 50% (w/v) Protein A/G PLUS agarose beads. To measure the activity of STEP, the immunocomplexes were suspended in 200 μl of assay buffer (in mM: 25 Hepes, pH 7.0, 20 $MgCl_2$, 0.1 PMSF) containing 15 mM p-NPP (cat# 20-106, Sigma Chemical) and incubated 60 min at 30°C under gentle stir. Phosphatase activity of STEP was measured in the clarified supernatants by colorimetric quantitation of the formation of p-nitrophenol at 410 nm using a spectrophotometer. For Western blot analysis, samples (40 μg of proteins) were resolved on 10% SDS–PAGE, and proteins were transferred to nitrocellulose (Schleicher and Schuell Bioscience Inc. Dassel, Germany). Blots were washed with TBS-0.05% Tween 20 (TTBS) and blocked with 5% BSA in TTBS for 2 h. Washed nitrocellulose filters were incubated overnight at 4° C with the following antibody: polyclonal anti-nonphospho STEP (Ser221) (D74H3) (cat# 5659; Cell Signaling, dilution 1:1000). After extensive washes in TTBS (approximately 10 for 10 min), the immunoreactive bands were detected by chemiluminescence coupled to peroxidase activity (ECL kit; cat# 32106, Thermo Scientific,) and quantified using a Bio-Rad ChemiDoc XRS system.

FMRP Immunoprecipitation

Brain extracts were prepared from cortex and hippocampus or striatum of WT and *Fmr1* KO male mice 3-4 weeks old, using RIPA buffer plus Protease inhibitor cocktail (Roche), Phosphatase inhibitor cocktails II and III (Sigma), 40 U/ml RNaseOUT (Invitrogen). Protein extracts were used for RNA-Immunoprecipitation (RIP) using a specific anti-FMRP antibody. Dynabeads previously saturated with 1% BSA in 1X PBS were incubated with anti-FMRP antibody or normal rabbit IgG (Santa Cruz Biotechnology) for 1 hour at room temperature (RT). The beads were then washed in a buffer (250 mM NaCl, 20 mM Tris-HCl pH 7.4, 10 mM MgCl2 and 0.1% Triton X-100), the protein extract added to the Dynabeads and incubated for 1-2 hours at 4°C. RNAs were eluted in TRIzol (Invitrogen). Upon RIP, the RNAs co-immunoprecipitated were extracted and analyzed by RT-qPCR, using the

StepOne Plus 7500 instrument (Life technologies) and specific oligonucleotides designed to amplify the RNAs of interest (Table S2).

Saturation binding experiments

Striatum, cortex, and hippocampus from WT and *Fmr1* KO mice (2-3 months of age) were homogenized with a Polytron in 50 mM Tris HCl, pH 7.4, and centrifuged for 20 min at 40.000 g. The resulting pellets were homogenized again and incubated in the presence of 2 IU of adenosine deaminase for 20 min at 37 °C to eliminate endogenous adenosine. The suspensions were then centrifuged for 20 min at 40.000 g and the pellets resuspended in 50 mM Tris HCl, pH 7.4 for the binding experiments.

Saturation binding experiments were carried out by using the $A_{2A}R$ antagonist [^{3}H]-ZM241385 (specific activity 27 Ci/mmol, Biotrend) as radioligand. Membranes were incubated in 50 mM Tris HCl, pH 7.4 with different concentrations (0.1-20 nM) of radioligand for 60 min at 4 °C. Nonspecific binding was determined in the presence of 10 μM ZM241385. At the end of the incubation time, bound and free radioactivities were separated by filtering the assay mixture through Whatman GF/B glass fiber filters in a Brandel cell harvester (Brandel). Filter bound radioactivity was counted in a Perkin Elmer 2810TR scintillation counter (Perkin-Elmer).

Statistics

Results from electrophysiological experiments were expressed as mean ± SEM (independent number of slices). The number of animals from which the slices have been obtained is reported for each data set in the Results. Data from the behavioral, dendritic spine, and testis weight evaluations were presented as mean ± SEM (independent number of mice). Results from *in vitro* biochemistry experiments were expressed as a percentage of control, which was considered as 100%, and as mean ± SEM values of at least 3 independently performed experiments (each independent experiment corresponds to an independent tissue lysate preparation from each mouse, thus n indicates the number of mice). Statistical analysis of the electrophysiology, behavioral, dendritic spine, biochemistry, and testis weight data was performed by using Mann-Whitney test. For STEP activity analysis results are expressed as mean ± SEM values of at least 4 independently performed experiments (each independent experiment corresponds to an independent tissue lysate preparation). Results from immunoprecipitation and RT-qPCR were expressed as mean ± SEM, and p-values were calculated

by Student's t-test. Statistical analysis for behavioral evaluation was performed separately on male and female *Fmr1* KO animals, and then results combined in case no differences were observed. Dissociation equilibrium constants for saturation binding, affinity, or KD values, as well as the maximum densities of specific binding sites (Bmax), were calculated for a system of one- or two-binding site populations using a non-linear curve fitting. All binding data are reported as mean ± SEM of at least 3 independent experiments. Differences between the groups were analyzed with Student's t-test. A p-value <0.05 was considered to indicate a significant difference. Variances between groups were similar. Statistical tests were two-sided. Statistical test for the evaluation of KW6002 on macroorchidism was one-sided since no increase of testis weight has been reported in the preclinical and clinical characterization of the drug as a Parkinson's disease medication. All statistical analyses and curve fittings were obtained using GraphPad Prism software (RRID: SCR_002798). We did not apply any inclusion or exclusion criteria to samples or animals.

Supplemental Figures and Tables

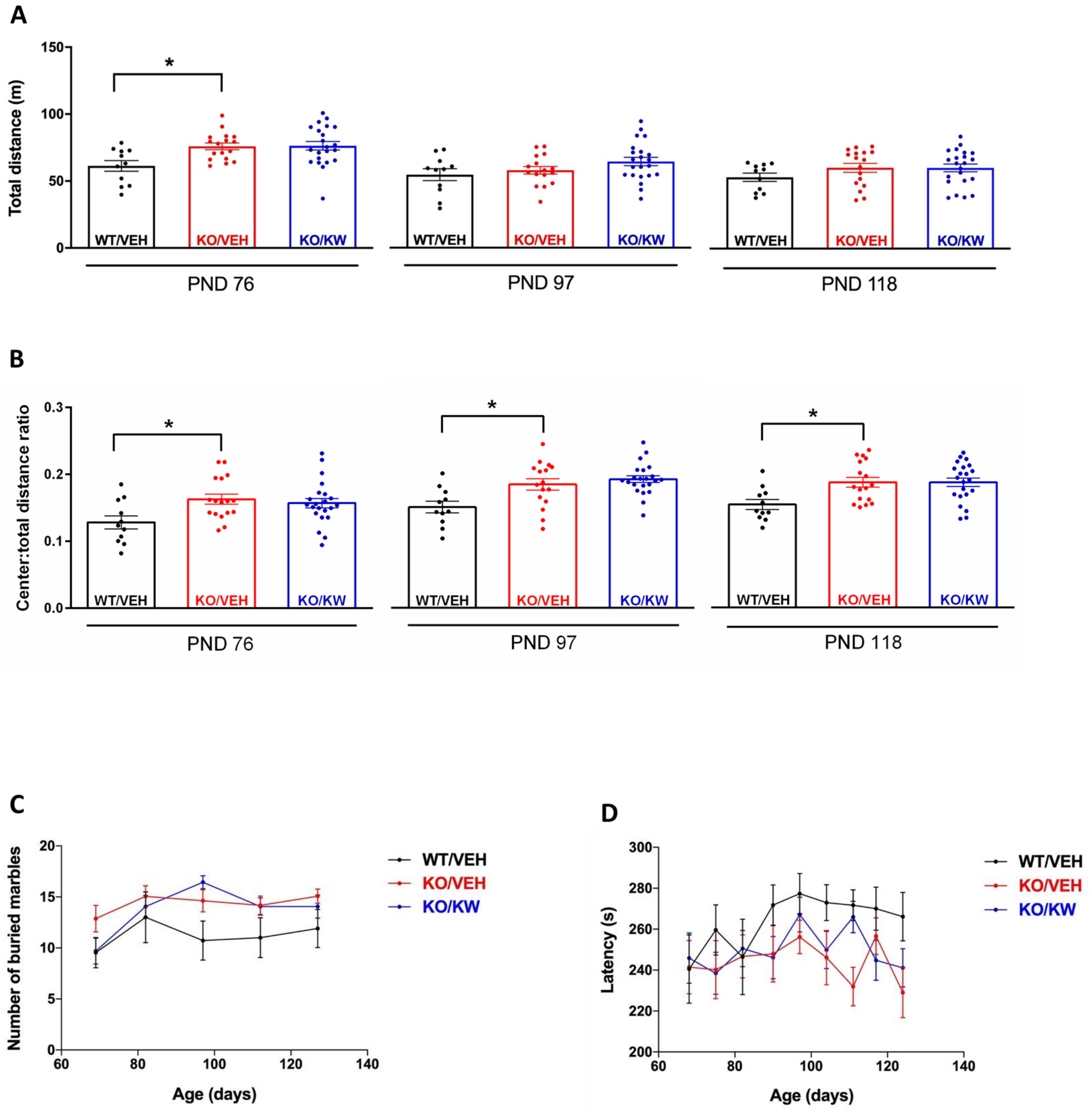

Figure S1. Chronic KW6002 treatment did not affect hyperactivity, stereotyped behavior, and motor coordination of *Fmr1* KO mice. a The experimental groups were: WT VEH mice (n=11; 6 males and 5 females), *Fmr1* KO VEH mice (n=16; 8 males and 8 females) and *Fmr1* KO KW mice (n=22; 12 males and 10 females). Locomotor activity: total distance traveled by KO/VEH mice was found increased in comparison to WT/VEH mice only at PND 76 (75.95 ± 2.51 vs. 61.3 ± 4.03, respectively, *p < 0.01, Mann-Whitney test) but the treatment with KW6002 did not affect this parameter (75.95 ± 2.51 in KO/VEH vs. 76.35 ± 3.19 in KO/KW, p = 0.7903). **b** KO/VEH mice spent

a greater proportion of time in the center of the open field compared to WT/VEH mice as expressed by the center/total distance ratio at all the time points examined; in fact, significant increases in the ratio were found in KO/VEH mice at PND 76 (0.163 ± 0.0076 vs. 0.13 ± 0.0098, *$p < 0.05$, Mann-Whitney test), at PND 97 (0.184 ± 0.0085 vs. 0.150 ± 0.0086, *$p < 0.05$, Mann-Whitney test) and PND 118 (0.187 ± 0.0073 vs. 0.153 ± 0.0075, *$p < 0.05$, Mann-Whitney test). Again, such a parameter was not affected by chronic treatment with KW6002. Then, in agreement with the previous observations[3], we confirmed that *Fmr1* KO mice spend a greater portion of their distance traveled in the center area of the open field compared to control mice, being this feature, which is considered an index of lower anxiety, contrary to the FXS clinical phenotype[3]. As for the inability of KW6002 to prevent such an effect, it should be considered that $A_{2A}R$ antagonists do not affect the anxiety parameters in the elevated plus-maze test[4, 5]. Interestingly, furthermore, in spontaneously hypertensive rats (SHR), a widely used animal model useful to study the neurodevelopmental disorder ADHD, chronic blockade of $A_{2A}R$ was able to improve NOR performance[6], while being ineffective on hyperlocomotion and low anxiety behavior[7]. **c** Stereotyped behavior: the effect of treatment with KW6002 on stereotyped behavior was evaluated in marble-burying tests, but, at least in our experimental conditions, we did not observe any significant difference between KO/VEH and WT/VEH mice in terms of buried marbles and then it has not been possible to deduce the effect of KW6002. It should be taken into account that the reliability of the marble-burying test in mimicking obsessive-compulsive disorder (OCD) or anxiety is strongly debated; it has been observed that some anxiolytic compounds with no human anti-compulsive effects consistently pass this assay while some known anti-compulsive drugs consistently fail[8]. **d** Rotarod test: chronic treatment with KW6002 did not affect motor coordination and skill learning of animals assessed in the accelerating rotarod test. Motor coordination was almost comparable in *Fmr1* KO and WT mice and, anyway, chronic drug treatment did not affect the rotarod performance. This is in line with the lack of effect on locomotor function already reported for KW6002, up to 10 mg/kg per day[9].

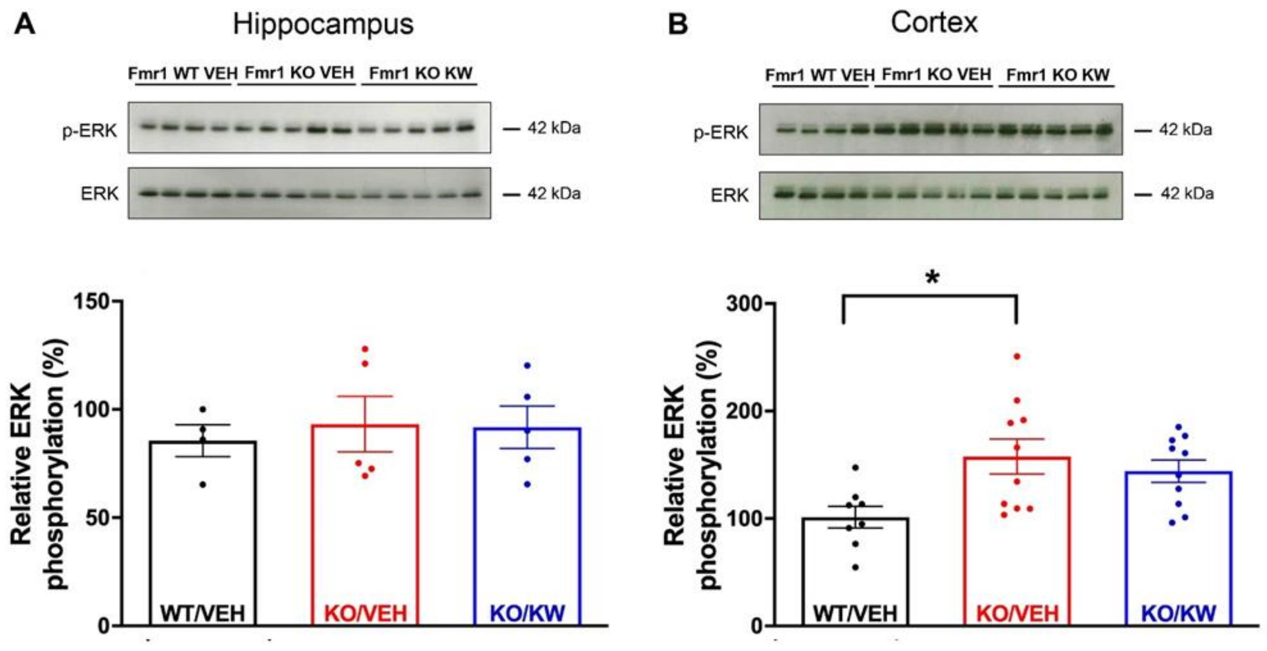

Figure S2. Chronic KW6002 treatment did not affect ERK1/2 signaling. a The levels of ERK1/2 phosphorylation were analayzed in the hippocampus, and (**b**) cortex from WT and *Fmr1* KO mice treated with KW6002 or vehicle. **a** Upper panel, representative Western blot showing ERK1/2 and p-ERK1/2 protein levels in the hippocampus. Lower panel, histogram shows the quantification of ERK1/2 phosphorylation. **b** Upper panel, representative Western blot showing ERK1/2, p-ERK1/2 protein levels in the cortex. Lower panel, histogram shows ERK1/2 phosphorylation levels in KO/VEH cortex (157.7 ± 16.24; n = 10) compared to WT/VEH mice (101.2 ± 10.08; n = 8; *p < 0.05 vs WT, Mann-Whitney test). The treatment with KW6002 did not reduce ERK1/2 hyper-phosphorylation in *Fmr1* KO mice (144 ± 10.36; n = 10). **(a, b)** the histograms indicate the % of phosphoproteins normalized to respective total protein levels. The ERK signaling pathway is found hyper-responsive in *Fmr1* KO mice[10] and increased in the frontal cortex of FXS patients[11]. Our biochemistry data confirm that ERK phosphorylation level is significantly higher specifically in the cortex of vehicle-treated *Fmr1* KO animals compared to WT mice, but the chronic blockade of $A_{2A}R$ did not affect this hyper-phosphorylation.

Table S1

List of specific oligonucleotides designed to amplify the RNAs of interest in the FMRP-Immunoprecipitation experiment.

mHprt1	CAGCCCCAAAATGGTTAAGGTTGC
	TCCAACAAAGTCTGGCCTGTATCC
mCamK2α	GTGCTGGCTGGTCAGGAGTATGC
	CTTCAACAAGCGGCAGATGCGGG
$mA_{2A}R$	GTCCTGGTCCTCACGCAGAGTTCCATC
	AGCCATTGTACCGGAGTGGA
mH3	ACTGACTGTTCACAGACC
	CCATCCCTTCTGCGTATTAG
mMap1b	TTCCAGGACAAAAGATTCTTC
	GGCTTCATCTGAAGGGTTGA

Table S2

Affinity (KD, nM) and density (Bmax, fmol/mg protein) of A_{2A}Rs in striatum, cortex, and hippocampus obtained from WT or Fmr1 KO mice.

	**WT**	**Fmr1 KO**
	[^{3}H]-ZM241385<br>K_D (nM)<br>Bmax (fmol/mg protein)	[^{3}H]-ZM241385<br>K_D (nM)<br>Bmax (fmol/mg protein)
Striatum	0.97 ± 0.09<br>1154 ± 92	0.93 ± 0.08<br>1123 ± 73
Cortex	1.26 ± 0.11<br>112 ± 10	1.32 ± 0.09<br>117 ± 8
Hippocampus	1.64 ± 0.12<br>48 ± 4	1.71 ± 0.15<br>52 ± 5

Data are expressed as the mean ± SEM of 3 independent experiments performed in duplicate.

Affinity and density of A_{2A}Rs are comparable in the brain areas of *Fmr1* KO and WT mice. Saturation binding experiments performed with the antagonist radioligand [^{3}H]-ZM241385 revealed that no differences in A_{2A}R affinity and density were observed in striatum, cortex, and hippocampus from WT and *Fmr1* KO mice in basal conditions.

References

1. Irwin, S.A. et al. Dendritic spine and dendritic field characteristics of layer V pyramidal neurons in the visual cortex of fragile-X knockout mice. *Am. J. Med. Genet.* **111**, 140-146 (2002).

2. Chiodi, V. et al. Cocaine induced changes of synaptic transmission in the striatum are modulated by adenosine A_{2A} receptors and involve the tyrosine phosphatase STEP. *Neuropsychopharmacology* **39**, 569-578 (2014).

3. Kazdoba, T.M., Leach, P.T., Silverman, J.L., Crawley, J.N. Modeling fragile X syndrome in the Fmr1 knockout mouse. *Intractable Rare Dis. Res.* **3**, 118-133 (2014).

4. El Yacoubi, M., Ledent, C., Parmentier, M., Costentin, J. & Vaugeois, J.M. The anxiogenic-like effect of caffeine in two experimental procedures measuring anxiety in the mouse is not shared by selective A(2A) adenosine receptor antagonists. *Psychopharmacology (Berl).* **148**, 153-163 (2000).

5. Prediger, R.D., Batista, L.C. & Takahashi, R.N. Adenosine A1 receptors modulate the anxiolytic-like effect of ethanol in the elevated plus-maze in mice. *Eur. J. Pharmacol.* **499**, 147-154 (2004).

6. Pires, V.A., Pamplona, F.A., Pandolfo, P., Prediger, R.D. & Takahashi, R.N. Chronic caffeine treatment during prepubertal period confers long-term cognitive benefits in adult spontaneously hypertensive rats (SHR), an animal model of attention deficit hyperactivity disorder (ADHD). *Behav. Brain Res.* **215**, 39-44 (2010).

7. Pandolfo, P., Machado, N.J., Köfalvi, A., Takahashi, R.N. & Cunha RA. Caffeine regulates frontocorticostriatal dopamine transporter density and improves attention and cognitive deficits in an animal model of attention deficit hyperactivity disorder. *Eur. Neuropsychopharmacol.* **23**, 317-328 (2013).

8. Albelda, N. & Joel, D. Animal models of obsessive-compulsive disorder: exploring pharmacology and neural substrates. *Neurosci. Biobehav. Rev.* **36**, 47–63 (2012).

9. Orr, A.G. et al. Istradefylline reduces memory deficits in aging mice with amyloid pathology. *Neurobiol. Dis.* **110**, 29-36 (2018).

10. Osterweil, E.K., Krueger, D.D., Reinhold, K. & Bear, M.F. Hypersensitivity to mGluR5 and ERK1/2 leads to excessive protein synthesis in the hippocampus of a mouse model of fragile X syndrome. *J. Neurosci.* **30**, 15616-15627 (2010).

11. Wang, X. et al. Activation of the extracellular signal-regulated kinase pathway contributes to the behavioral deficit of fragile x-syndrome. *J. Neurochem.* **121**, 672-679 (2012).
